# Chinese medicine combined with EGFR-TKIs in EGFR-mutated advanced NSCLC: a meta-analysis of efficacy and safety

**DOI:** 10.3389/fonc.2026.1942222

**Published:** 2026-10-12

**Authors:** Xiaohong Li, Die Qian, Shuai Liu, Mingmin Na, Bihong Dai, Chunping Wan

**Affiliations:** 1 Department of Scientific Research, The Fifth Affiliated Hospital of Yunnan University of Chinese Medicine, Chuxiong, China; 2 First Clinical Medical College, School of Pharmacy and School of Basic Medicine, Yunnan University of Chinese Medicine, Kunming, China

**Keywords:** efficacy, epidermal growth factor receptor tyrosine kinase inhibitors (EGFR-TKI), non-small cell lung cancer (NSCLC), safety, traditional Chinese medicine (TCM)

## Abstract

**Objective:**

For advanced EGFR-mutant non-small cell lung cancer (NSCLC), epidermal growth factor receptor tyrosine kinase inhibitors (EGFR-TKI) therapy is the primary treatment, but drug resistance and side effects reduce its effectiveness. Integrating Chinese and Western medicine is a key focus in cancer treatment. This study evaluates the clinical benefits of combining traditional Chinese medicine (TCM) with EGFR-TKI, summarizing TCM syndromes and medication traits.

**Methods:**

We searched six databases for relevant clinical studies. A meta-analysis assessed progression-free survival (PFS), disease control rate (DCR), and adverse reactions, with subgroup analysis by EGFR mutation type. A TCM syndrome network was created to examine syndrome distribution and core prescription patterns.

**Results:**

TCM combined with EGFR-TKIs significantly improved PFS (HR = 0.70, 95%CI 0.65–0.76, *p* < 0.00001, I² = 0%), benefiting patients with Exon 19 deletion or L858R mutation equally (*p* = 0.75). The combination therapy increased DCR (RR = 1.19, 95% CI 1.01–1.40, *p* = 0.04), with substantial heterogeneity (I² = 89%), and reduced targeted-agent-related adverse reactions. Qi-Yin and Lung-Spleen deficiency were the dominant TCM syndromes, managed via Qi-tonifying, Yin-nourishing and spleen-strengthening regimens.

**Conclusion:**

Integrating TCM with EGFR-TKI enhances tumor control and treatment safety in advanced NSCLC patients with EGFR mutations, showing consistent efficacy across various mutation types. This approach, which considers clinical syndrome differentiation and medication patterns in Chinese medicine, offers evidence-based guidance for personalized treatment. This review was performed following the PRISMA 2020 guidelines.

**Systematic Review Registration:**

https://www.crd.york.ac.uk/PROSPERO/view/CRD420261398486, identifier CRD420261398486.

## 1 Introduction

Lung cancer, the most prevalent and lethal malignant tumor globally, is primarily divided into non-small cell lung cancer (NSCLC) and small cell lung cancer (SCLC) (1). Approximately 85% of all lung cancer cases are NSCLC, which is the leading pathological subtype (2). Epidermal growth factor receptor (EGFR) sensitive mutations serve as critical driver gene mutations in NSCLC, with an overall incidence of approximately 30% among NSCLC patients (3). Exon 19 deletions and exon 21 L858R mutations are the classic EGFR activating mutations, making up 85% to 90% of all mutations in the EGFR kinase domain (4). Numerous epidemiological studies and meta-analyses have substantiated the existence of significant racial disparities in EGFR mutation rates. Among patients with NSCLC in predominantly East Asian populations, the EGFR mutation rate ranges from 38% to 50%, which is markedly higher compared to European (14%), North American (15%), and Oceanian (12%) populations (5). This disparity is especially evident in individuals with lung adenocarcinoma, females, and those with no history of smoking.

EGFR mutations play a critical role in determining the sensitivity of advanced NSCLC to EGFR tyrosine kinase inhibitor (TKI) therapy (6). EGFR-TKIs have emerged as the first-line standard treatment for these patients due to their substantial progression-free survival (PFS) benefits and favorable safety profiles (7, 8). Nevertheless, the majority of patients eventually develop acquired resistance during treatment, and the 5-year survival rate for individuals with metastatic EGFR-mutated lung cancer remains approximately 15% (9). This underscores the need for improved clinical outcomes and highlights the urgent necessity for the development of novel comprehensive treatment strategies. Traditional Chinese Medicine (TCM), with its long-standing history in the holistic management of NSCLC, offers potential benefits. By adhering to the principle of syndrome differentiation, TCM interventions can significantly enhance patients’ quality of life (10). Clinical evidence suggests that the integration of TCM with EGFR-TKIs constitutes a highly valuable therapeutic approach.

This study concentrates on patients with advanced NSCLC who have been confirmed to possess EGFR mutations. It systematically evaluates the clinical efficacy and safety of integrating TCM with EGFR-TKIs. Additionally, the study organizes and synthesizes the characteristics of TCM formulations and their compatibility principles.

## 2 Methods

This study has been registered with the PROSPERO database (CRD 420261398486) and is publicly available at https://www.crd.york.ac.uk/PROSPERO/view/CRD420261398486. The design and reporting of this systematic review follow the PRISMA 2020 statement. The completed PRISMA 2020 checklist is provided as a supplementary file. The certainty of evidence was assessed using the GRADE framework.

### 2.1 Search strategy

Literature screening was performed in six databases from their inception through May 18, 2026. The included data were retrieved from six databases; detailed search protocols can be found in **Supplementary Table 1**. We applied language restrictions to include only English and Chinese-language publications. Peer-reviewed journal articles constituted our primary source; we also searched thesis databases for grey literature records, although dissertations with severe methodological flaws or incomplete outcome data, as well as conference abstracts lacking full-text data, were excluded. We searched CNKI and Wanfang for Chinese dissertations, screened trial registries (ChiCTR, ClinicalTrials.gov), and manually checked the reference lists of included studies and relevant systematic reviews.

### 2.2 Study eligibility

Studies were included based on the following requirements: (1) original articles were restricted to observational studies and RCTs; (2) all subjects were over 18 years of age, with pathologically or cytologically verified inoperable stage III–IV NSCLC and detectable sensitive EGFR mutations; (3) patients in the TKIs plus TCM group received combination therapy of EGFR-TKIs and TCM via oral administration, external application or intravenous infusion. The main EGFR-TKIs adopted in primary studies were gefitinib, erlotinib and icotinib. (4) Literature that has been formally published following peer review.

Exclusion criteria were: (1) articles that were not original research, such as literature reviews, editorial comments and previous meta-analyses; (2) study protocols; (3) full text not available; (4) NSCLC patients without EGFR mutations; (5) studies not using TCM combined with EGFR-TKI treatment.

### 2.3 Data extraction

Two reviewers (X.L, D.Q) independently screened the literature and extracted data using Excel. The data included: (1) Study details: first author, year, and design; (2) NSCLC patient information: population size, age, and EGFR mutation details (exon 19 deletion, exon 21 L858R); (3) EGFR-TKI types, TCM syndromes, and Chinese herbal medicine prescriptions; (4) Study endpoints: PFS and DCR, (5) Adverse reaction rates to assess safety in NSCLC patients.

### 2.4 Assessment of bias risk

Risk of bias assessment was performed for included studies; the Cochrane RoB tool was adopted for RCTs and the NOS scale for observational studies. The quality assessment was independently conducted by two investigators (X.L and S.L), with any disagreements resolved by two other reviewers (D.Q and M.N).

### 2.5 Statistical analyses

Statistical analyses were conducted using Review Manager 5.3. Pooled estimates for the primary outcome, with 95% confidence intervals, were calculated using a random-effects model. Inter-study heterogeneity was assessed with the I² statistic, where I² ≥ 50% indicated significant heterogeneity, and extreme heterogeneity (I² = 75%–100%) precluding quantitative pooling for further heterogeneity source exploration. Hazard ratios and risk ratios, with 95% CIs, were used for PFS and DCR data synthesis. Subgroup analysis and meta-regression identified heterogeneity sources. Sensitivity analysis checked outcome robustness, while funnel plots and Egger’s test evaluated publication bias.

Data processing and statistical analysis of TCM were carried out using R software (version 4.5.2). Descriptive statistics, correlation clustering, complex network analysis, and syndrome-drug association mining were performed using the Tidyverse, ComplexHeatmap, circlize, dendextend, cluster, arules, arulesViz, ggraph, tidygraph, and igraph packages.

## 3 Results

### 3.1 Selecting and identifying studies

The literature screening process is illustrated in **Figure 1**. The search period was from database inception through May 18, 2026. Out of the 1,163 articles found, 352 were repeated. After reviewing the titles and abstracts, 574 articles were deemed irrelevant, and 184 were classified as meta-analyses or reviews and subsequently excluded. A full-text examination revealed that 14 articles involved animal studies, 12 were study protocols, and 9 had incomplete data. Finally, 17 articles were incorporated into the meta-analysis, among which 5 studies conducted a comparative analysis of exon 19 deletion and exon 21 mutations for NSCLC (11–27).

**Figure 1:**
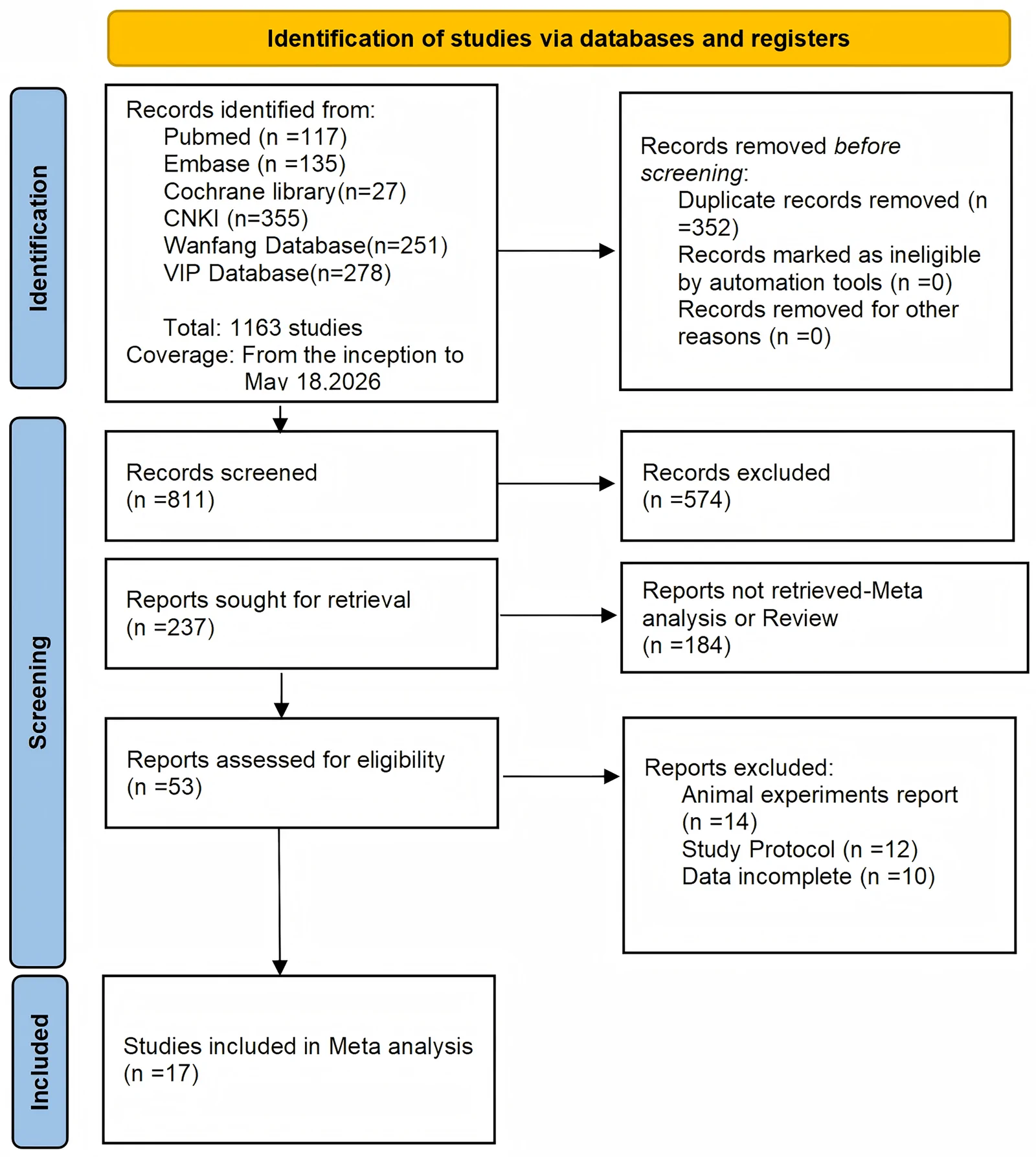
. A diagram illustrating the process of selecting articles.

### 3.2 General characteristics of included literature

Out of the 17 studies that were part of the analysis: 4 were cohort studies, and 13 were RCTs. The total sample comprised 2,088 patients diagnosed with NSCLC, of whom 1,101 received treatment with TCM in combination with EGFR-TKIs, while 987 patients received EGFR-TKI treatment alone. Of the included studies, 10 specifically reported the number of patients with EGFR mutations, distinguishing between exon 19 deletions and exon 21 L858R mutations. In the TCM+EGFR-TKI treatment cohort, 397 patients exhibited exon 19 deletions and 407 patients exhibited exon 21 L858R mutations. Conversely, in the EGFR-TKI treatment cohort, 377 patients had exon 19 deletions and 387 patients had exon 21 L858R mutations. Regarding the types of EGFR-TKI treatments administered, 1 study employed the third-generation inhibitor Osimertinib, another utilized the second-generation inhibitor Afatinib, and 16 studies employed first-generation EGFR-TKIs, including Gefitinib, Erlotinib, and Icotinib. **Supplementary Table 2** contains detailed characteristics of the studies included.

The study encompassed 33 prescriptions, incorporating 95 medicinal substances, with a cumulative usage frequency of 316 instances. The medicinal effects were predominantly categorized into tonic drugs, heat-clearing drugs, and those facilitating phlegm resolution, cough relief, and asthma alleviation. The Traditional Chinese Medicine syndromes were primarily classified into eight types: Deficiency of Qi and Yin, Lung-Spleen Deficiency, Heat-Toxic Obstruction of the Lung, Phlegm-Dampness-Stasis, Qi Deficiency, Yin Deficiency, Qi Deficiency and Blood Stasis, and Yin Deficiency with Lung Dryness (28).

### 3.3 Primary Outcomes: PFS

A total of 13 studies initially reported PFS; however, data from 2 studies were unavailable and thus excluded from the analysis. Consequently, 11 studies were included in the final analysis. The findings indicated that the PFS in the TCM combined with EGFR-TKI group was significantly greater than that in the EGFR-TKI group alone (HR, 0.70; 95% CI, 0.65–0.76; *p* < 0.00001), with no significant heterogeneity observed among the studies (I² = 0%) (**Figure 2**).

**Figure 2:**
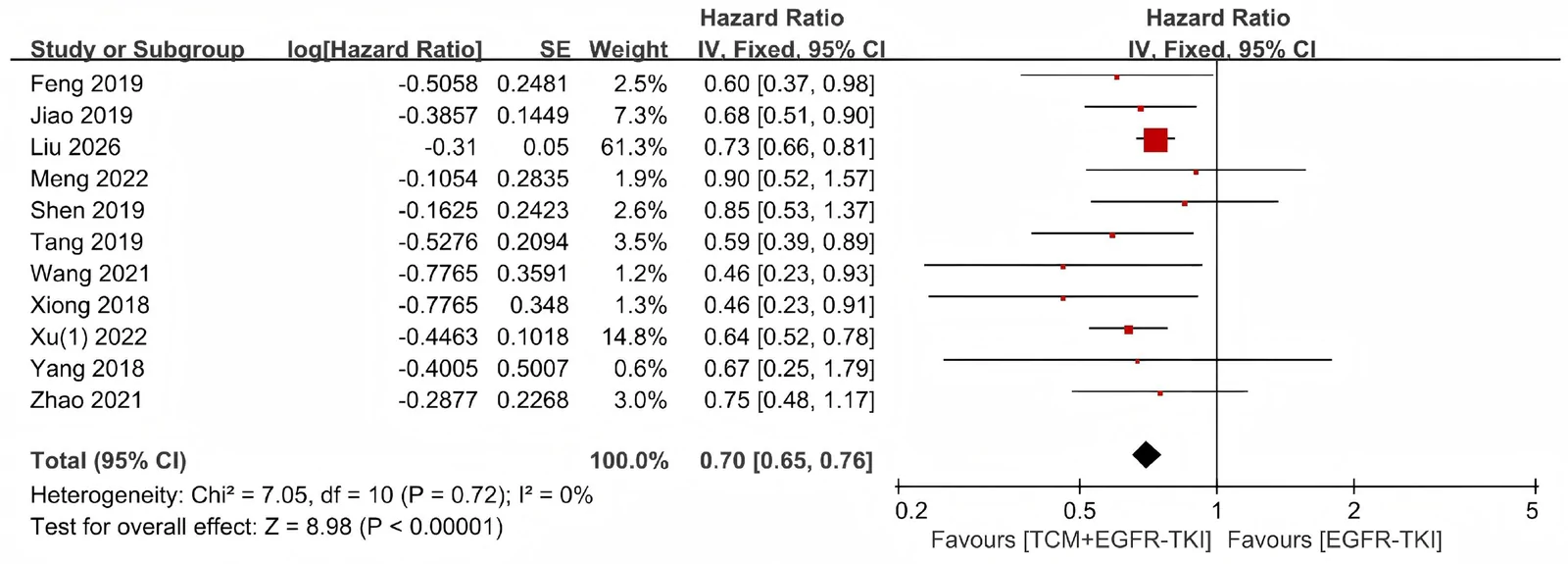
. A forest plot comparing the effects of TCM+EGFR-TKI versus EGFR-TKI alone on PFS in patients with advanced NSCLC and EGFR mutations.

In the subgroup analysis of PFS, five studies reported PFS outcomes for patients with EGFR mutations involving exon 19 deletions and exon 21 L858R mutations; however, one study did not provide data for exon 19 deletions. The analysis demonstrated that the combination of TCM with EGFR-TKI therapy significantly extended PFS compared to EGFR-TKI therapy alone in both the exon 19 deletion subgroup (HR = 0.64; 95% CI, 0.51-0.79; *p* < 0.0001) and the exon 21 L858R mutation subgroup (HR = 0.60; 95% CI 0.46-0.78; *p* = 0.0001). According to the test for subgroup differences, there was no statistically significant variation in treatment effects between the two mutation subtypes (*p* = 0.75), indicating that the PFS benefit associated with the addition of TCM is consistent across different EGFR mutation subtypes, with no particular subtype exhibiting a superior benefit (**Figure 3**).

**Figure 3:**
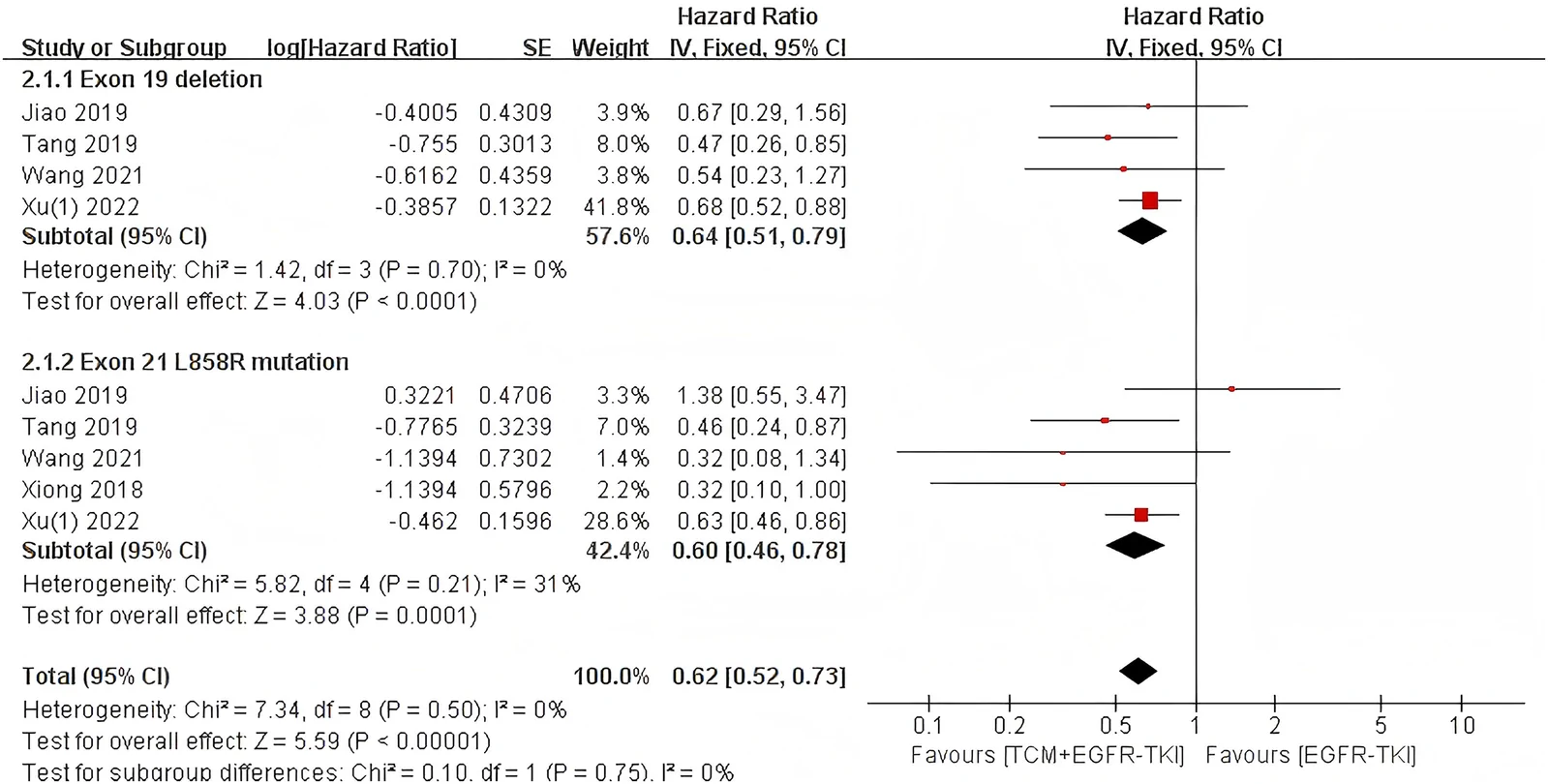
Comparison of PFS in advanced NSCLC patients with exon 19 deletion and exon 21 L858R mutation using a forest plot for TCM+EGFR-TKI versus EGFR-TKI treatments.

### 3.4 Secondary outcomes: DCR

The analysis of eight studies shows that combining TCM with EGFR-TKI therapy can improve the DCR compared with TKI monotherapy (RR = 1.19, 95% CI 1.01–1.40, *p* = 0.04, **Figure 4**). Despite initial high heterogeneity (I² = 89%), excluding two studies by Jiao. 2019 and Zhao. 2021 (12, 26), the heterogeneity decreased significantly, while maintaining statistically significant benefits (*p* = 0.001, I² = 34%, **Supplementary Figure 1**). These findings imply that adjuvant TCM may have the potential to augment the therapeutic effect of EGFR-TKIs, which provides preliminary evidence for exploring optimised treatment strategies in patients with EGFR-mutant NSCLC.

**Figure 4:**
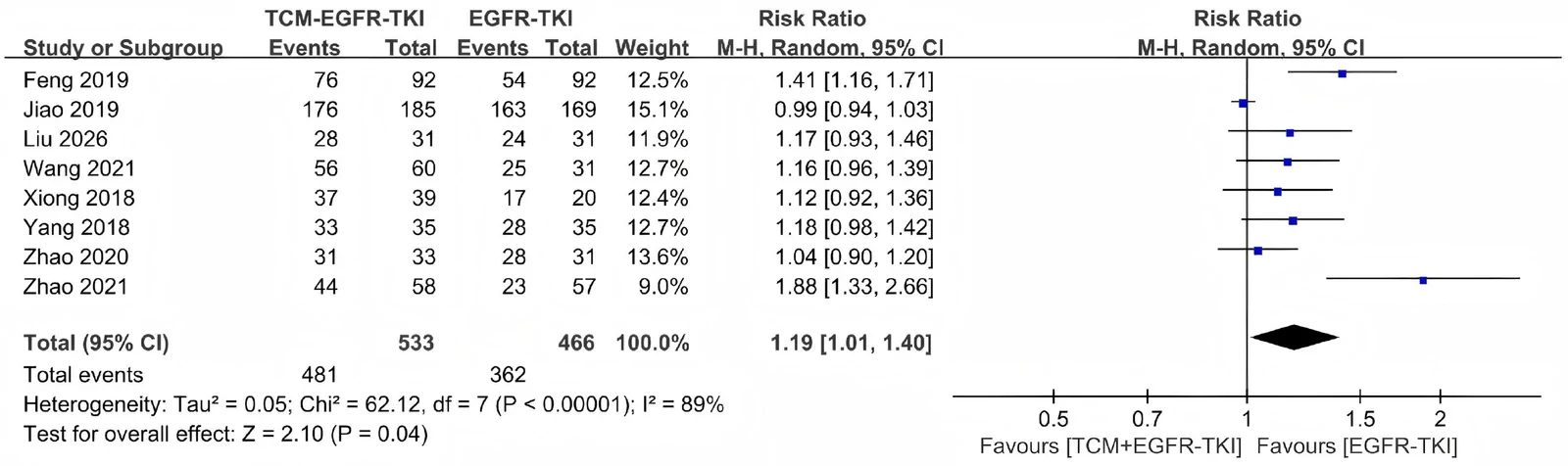
Forest plot comparing TCM+EGFR-TKI and EGFR-TKI treatments for DCR in patients with EGFR mutation-positive advanced NSCLC.

### 3.5 TCM+EGFR-TKI safety in NSCLC

Among the studies included in the analysis, 15 reported on the incidence of rash, with 1,022 cases observed in the TCM+EGFR-TKI group compared to 918 cases in the EGFR-TKI group. The findings indicated that the TCM+EGFR-TKI group significantly reduced the risk of rash (RR = 0.75; 95%CI, 0.61–0.92; *p* = 0.006), although there was moderate-to-high heterogeneity (I² = 67%). 12 studies assessed the incidence of diarrhea, reporting 744 cases in the TCM+EGFR-TKI group and 641 cases in the EGFR-TKI group. The results demonstrated a significant reduction in the risk of diarrhea in the TCM EGFR-TKI group (RR = 0.66; 95% CI, 0.51–0.86; *p* = 0.002), with moderate heterogeneity (I² = 59%). Seven studies examined liver function damage related to ALT/AST levels, with 392 cases in the TCM+EGFR-TKI group and 310 cases in the EGFR-TKI group. The analysis revealed that the TCM+EGFR-TKI group significantly reduced the risk of liver function damage (RR = 0.61; 95% CI, 0.41–0.91; *p* = 0.02), with no observed heterogeneity (I² = 0%). Additionally, five studies reported on the incidence of oral ulcers, with 257 cases in the TCM+EGFR-TKI group and 175 cases in the EGFR-TKI group, indicating a significant reduction in the risk of oral ulcers in the TCM+EGFR-TKI group (I² = 0%) (**Figure 5**).

**Figure 5:**
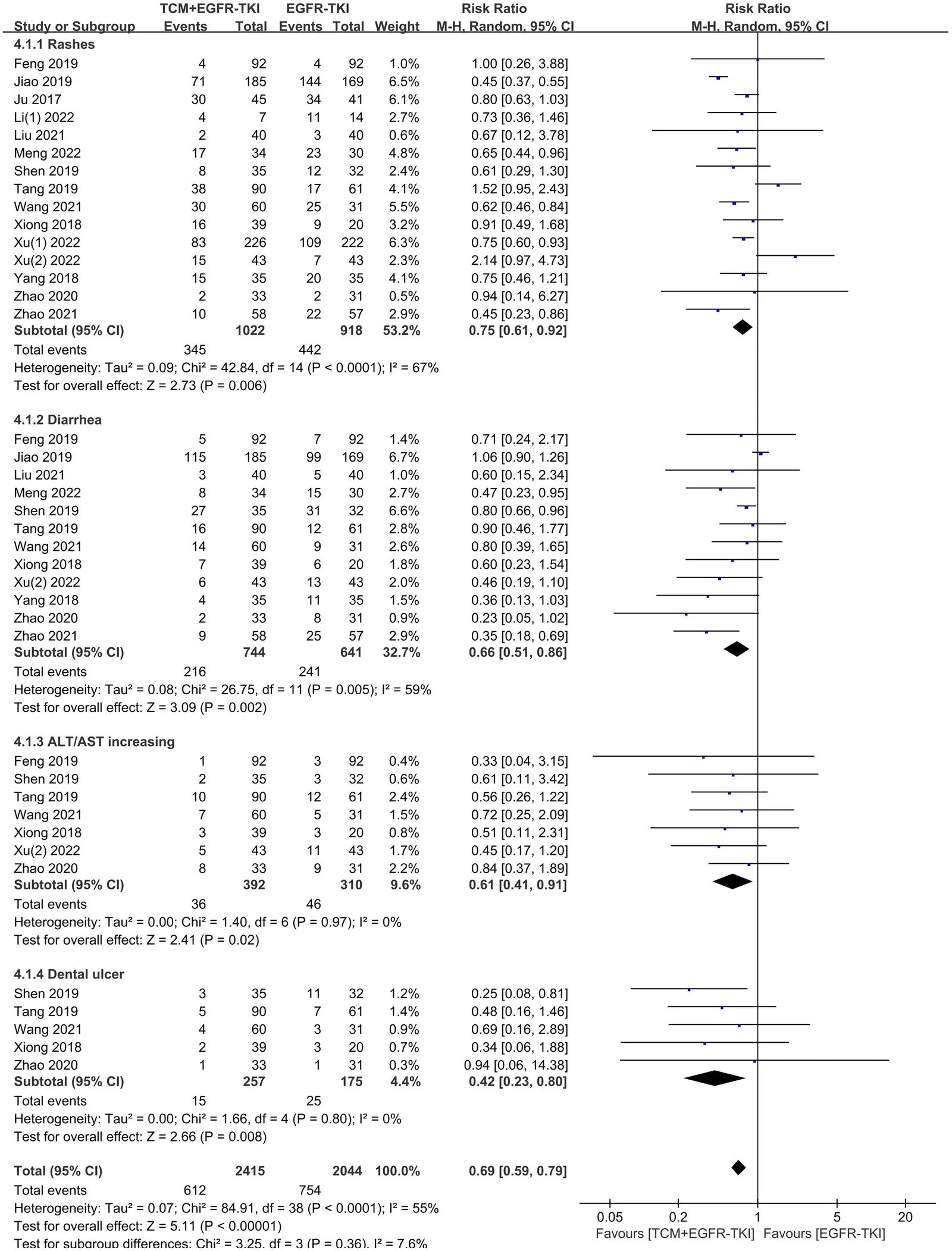
Forest plot comparing TCM+EGFR-TKI and EGFR-TKI treatments for adverse effects in patients with EGFR mutation-positive advanced NSCLC.

This meta-analysis suggests that integrating TCM with EGFR-TKI therapy is associated with a reduction in the risk of certain targeted therapy-related adverse events, including rash, diarrhea, liver function impairment, and oral ulcers, in patients with NSCLC. However, given the substantial heterogeneity observed across studies for some outcomes and the methodological limitations noted, these findings should be interpreted with caution. These results highlight the need for more rigorously designed, large-scale clinical trials to conclusively determine the role of TCM as an adjunctive therapy in mitigating EGFR-TKI-related toxicities.

### 3.6 The relationship between TCM syndromes and medicines

Frequency analysis of included Chinese herbal prescriptions revealed that the most commonly used herbs were *Astragali Radix* (Huangqi, 21 times, 6.6%), *Atractylodis Macrocephalae Rhizoma* (Baizhu, 20 times, 6.3%), followed by *Poria* (Fuling, 12 times, 3.8%) (**Figure 6A**). Regarding efficacy categories, the primary types identified were tonics, heat-clearing agents, and medications for resolving phlegm, relieving cough, and alleviating asthma (**Figure 6B**). Statistical analysis of the medicinal properties indicated that the medicines were predominantly cold in nature, sweet in taste, and primarily associated with the lung meridian (**Supplementary Figure 2**). Hierarchical cluster analysis categorized high-frequency pharmaceuticals into four distinct groups. The association rule bubble chart further elucidated numerous high-support, high-lift compatibility associations among heat-clearing and yin-nourishing agents, including *Radix Glehniae*, *Ophiopogon japonicus, Dendrobium, Herba Taraxaci, Prunella vulgaris*, and *Asparagus cochinchinensis* (**Figure 6C**). Additionally, complex network analysis revealed that *Astragalus, Atractylodes macrocephala, Ginseng, Poria, Herba Taraxaci*, and *Citrus reticulata* constitute the core high-frequency co-occurrence framework within the prescriptions (**Figure 6D**). Notably, the degree centrality for both *Astragalus* and *Atractylodes macrocephala* is 3, with weighted degrees of 38 and 37, respectively (**Figure 6E**).

**Figure 6:**
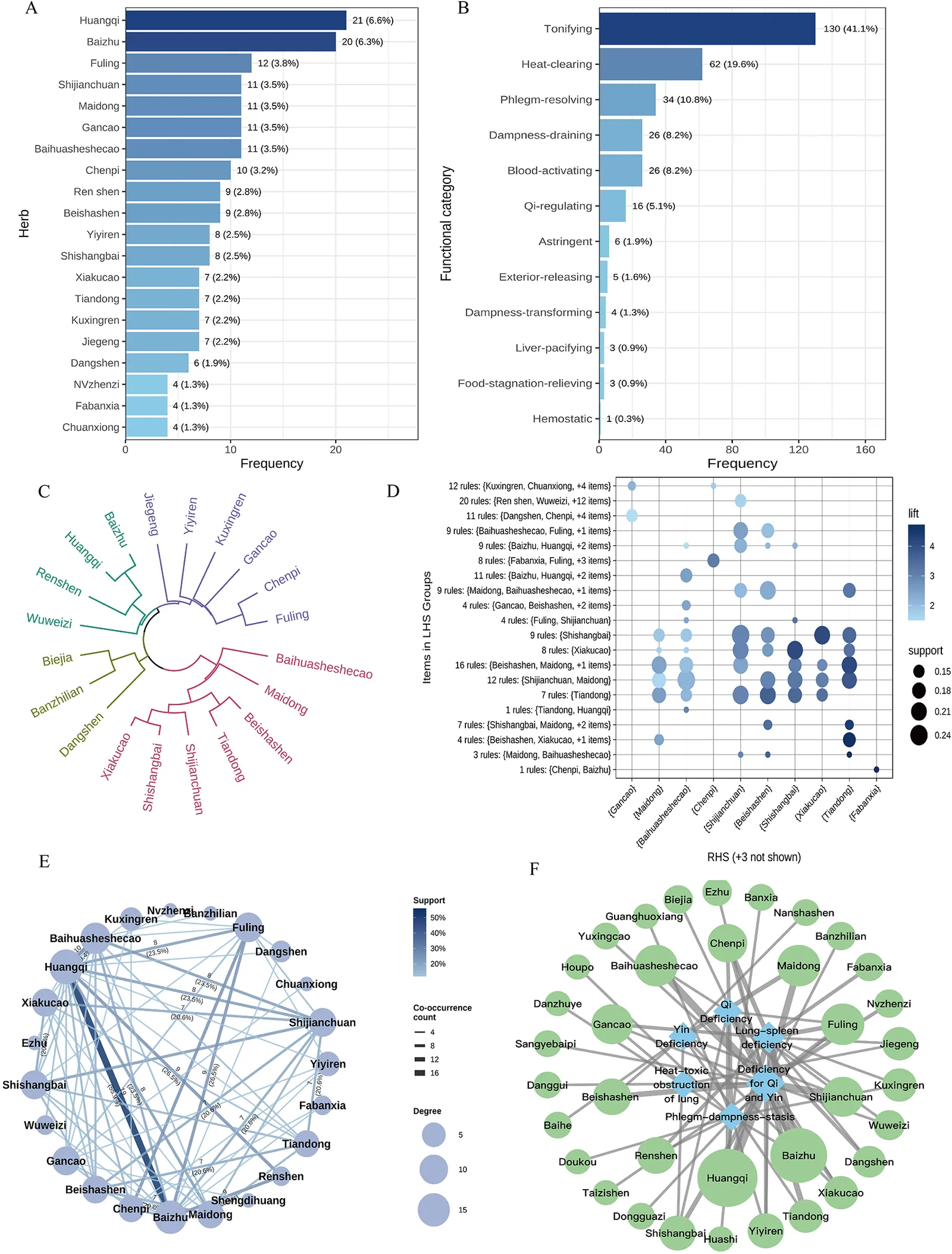
Compound composition and syndrome characteristics of included prescriptions. (**A**. Top 20 herbs by frequency; **(B)** Functional category distribution; **(C)** Hierarchical clustering dendrogram; **(D)** Association rules bubble plot; **(E)** Co-occurrence network of herbs; **(F)** Herb-functional category network.).

Syndrome-herb association network analysis demonstrated that Deficiency of Qi and Yin, and Lung-spleen Deficiency were the core syndromes, showing strong correlations with most high-frequency herbs. Key herbs across syndromes included *Astragali Radix, Atractylodis Macrocephalae Rhizoma, Poria, Ophiopogonis Radix, Citri Reticulatae Pericarpium* (Chenpi), *Glycyrrhizae Radix et Rhizoma* (Gancao), *Glehniae Radix* (Beishashen), and *Hedyotis Diffusae Herba* (**Figure 6F**).

Specific syndrome-based medication patterns were observed: Deficiency of Qi and Yin was treated with *Astragali Radix, Atractylodis Macrocephalae Rhizoma, Ophiopogonis Radix, Glehniae Radix*, *Schisandrae Chinensis Fructus* (Wuweizi), and *Codonopsis Radix* (Dangshen) to tonify qi and nourish yin; *Phlegm-dampness Stasis* with *Citri Reticulatae Pericarpium, Poria, Atractylodis Macrocephalae Rhizoma, Salvia Chinensis Herba* (Shijianchuan), *Coicis Semen* (Yiyiren), and *Pinelliae Rhizoma* (Banxia) to invigorate the spleen, resolve phlegm, and eliminate dampness and stasis; Lung-spleen Deficiency with *Astragali Radix*, *Atractylodis Macrocephalae Rhizoma, Codonopsis Radix, Poria*, and *Glycyrrhizae Radix et Rhizoma* based on the “reinforcing earth to generate metal” principle; and *Heat-toxic Obstruction* of the Lung with heat-clearing and detoxifying herbs such as *Hedyotis Diffusae Herba, Scutellariae Barbatae Herba* (Banzhilian), and *Houttuyniae Herba* (Yuxingcao) (**Supplementary Table 3**).

### 3.7 Study quality

We conducted a risk-of-bias evaluation for all included trials, and the overall results were presented in **Supplementary Figure 3** and **Supplementary Table 4**. Both forest plots and dot plots were adopted to demonstrate and analyze the bias risk of each enrolled study. The GRADE framework was applied to rate the certainty of evidence for critical and important outcomes The overall evidence certainty varied across different endpoints. Detailed GRADE-based evidence-certainty assessment for each outcome is shown in the **Supplementary Table 5**.

### 3.8 Sensitivity analysis

Weight-based sensitivity analysis was performed for the 11 included studies. The study by Liu et al. contributed up to 61.34 % of total weight under random-effects model, indicating strong influence on pooled estimate. The pooled HR fluctuated from 0.66 to 0.70 with overlapping 95%CI, and the direction of effect remained unchanged, which supported the robustness of our main finding. Given the dominant weight of this single study, leave-one-out sensitivity analysis was further conducted. (**Supplementary Figures 4**, **5**).

### 3.9 Publication bias

Publication bias was assessed across the 11 studies included in the PFS analysis (**Supplementary Figure 6**). Visual inspection of the funnel plot showed a generally symmetrical distribution. Egger’s linear regression test yielded a non-significant intercept (*p* = 0.124; 95% CI: −3.399-0.478), indicating no statistically significant evidence of publication bias within this set of studies. It is important to note, however, that the statistical power of such tests is limited when based on a small number of studies, and the possibility of undetected publication bias cannot be ruled out. Therefore, while the current analysis does not indicate publication bias, this finding should be interpreted with caution. Publication bias was not assessed for outcomes with fewer than 10 studies due to insufficient power of statistical tests.

## 4 Discussion

### 4.1 Summary of meta-analysis findings

EGFR-TKIs have been established as the first-line standard treatment for advanced NSCLC with EGFR mutations (29). Nonetheless, challenges such as acquired resistance and drug-related adverse effects post-treatment not only compromise overall clinical efficacy but also significantly diminish patients’ quality of life (30). The integration of TCM with EGFR-TKIs has emerged as a prevalent comprehensive clinical treatment strategy (31). Consequently, this study conducts a systematic analysis of the clinical value and pharmacological characteristics of this integrated Chinese and Western medicine regimen, examining multiple dimensions including clinical efficacy, drug safety, the distribution of TCM syndrome types, and prescription patterns.

This meta-analysis demonstrates that combining TCM with EGFR-TKI treatment significantly prolongs PFS. Regarding the DCR, while initial pooled analysis indicated a statistically significant benefit for the combination therapy, substantial heterogeneity compromised the reliability of this finding. Consequently, a sensitivity analysis was performed. Excluding two studies (12 and 26) markedly reduced heterogeneity, suggesting these studies were the primary source of the original heterogeneity. The observed difference in heterogeneity between DCR and PFS may be attributable to the nature of the outcome measures. DCR can be susceptible to factors such as tumor assessment criteria, specific TCM prescriptions, intervention duration, and patient baseline characteristics. In contrast, PFS, as an objective time-to-event endpoint, exhibited greater consistency across studies. Although the initial DCR result demonstrated only borderline statistical significance, the sensitivity analysis supports the potential benefit of combination therapy for tumor control. Furthermore, the robust PFS findings suggest that the survival benefit conferred by TCM may arise not merely from short-term lesion control but also from mechanisms such as delaying TKI resistance and modulating the tumor immune microenvironment. Notably, moderate heterogeneity persisted for DCR even after excluding the two studies. This residual heterogeneity implies that factors including variations in TCM formulas, intervention duration, and the methodological quality of included studies may contribute to confounding effects. Therefore, interpretation of the DCR results warrants caution, and over-interpretation of its therapeutic efficacy should be avoided.

Subgroup analyses indicated that integrative therapy incorporating TCM was associated with improved PFS in both the EGFR Exon 19 deletion and Exon 21 L858R mutation subgroups, with no statistically significant difference in comparative efficacy observed between the two genetic subtypes. This conclusion is consistent with previous studies (31). From a molecular standpoint, EGFR-TKIs exert anti-tumour effects primarily through competitive binding to the tyrosine kinase domain of EGFR, wherein conformational differences between Exon 19 deletion and L858R mutations contribute to variations in TKI sensitivity. In contrast, Chinese herbal medicines exhibit multi-targeted anti-tumour mechanisms that extend beyond direct kinase inhibition. Preclinical studies have shown that certain herbal compounds can suppress key downstream proliferative pathways—such as PI3K/Akt/mTOR, RAS-MAPK signalling, and remodel the tumour immune microenvironment (32–34). These broad-spectrum regulatory effects are largely independent of the specific EGFR-mutation subtype, which may partly explain the consistent PFS benefit observed across both Exon 19 deletion and L858R subgroups in the present meta-analysis.

It should be noted, however, that this subgroup comparison was exploratory in nature and not pre-specified in the protocol. Importantly, the certainty of evidence for the L858R subgroup was graded as low, owing to the limited number of available studies and the presence of moderate statistical heterogeneity. Consequently, the findings regarding the comparable efficacy between the two mutation subtypes should be interpreted with caution. Further mechanistic investigations and large-scale prospective randomized controlled trials with pre-planned stratified analyses are warranted to validate these preliminary observations.

Safety analyses indicated that adjunctive therapy with TCM was associated with a reduced risk of EGFR-TKI-related adverse events, including rash, diarrhea, liver function impairment, and oral ulceration. This attenuating effect may be attributed to the mucosal protective properties or systemic immunomodulatory functions of herbal compounds. Preclinical models have demonstrated that formulations such as Sijunzi Decoction can protect the gastrointestinal mucosa, potentially ameliorating TKI-induced diarrhea and oral mucositis (35). Furthermore, TCM interventions are posited to modulate immune homeostasis, thereby mitigating the inflammatory-mediated adverse reactions commonly associated with EGFR-TKIs (36). However, none of the studies included in this meta-analysis reported objective biomarkers of mucosal integrity or systemic immunological parameters. Consequently, the observed reduction in adverse event incidence within the combination therapy group cannot be solely attributed to the pharmacodynamic detoxification effects of TCM. Future basic research and prospective clinical trials incorporating relevant biomarker assessments are warranted. Additionally, potential herb-drug pharmacokinetic interactions remain a relevant confounding factor that requires further investigation.

While our analysis suggests potential clinical benefits, the possibility of herb-drug pharmacokinetic interactions warrants careful consideration. The majority of EGFR-TKIs are primarily metabolized by hepatic CYP3A4/5 and serve as substrates for efflux transporters such as P-gp and BCRP. Concomitant administration of TCM interventions may alter the systemic exposure of TKIs, consequently impacting both efficacy and toxicity outcomes (37, 38). Although animal and *in vitro* studies have confirmed that certain TCM compounds and active constituents can modulate the pharmacokinetics of TKIs, high-quality human pharmacokinetic data for most commonly used anti-cancer TCM formulations are still lacking (35, 39). To date, no clinical trials have quantitatively assessed the actual effects of such interactions in patients with EGFR-mutant non-small cell lung cancer. Notably, none of the randomized controlled trials included in this meta-analysis monitored TKI plasma concentrations; thus, it remains impossible to distinguish whether the observed effects stem from pharmacokinetic interactions or direct pharmacodynamic anti-tumor actions. Clinicians should remain vigilant regarding potential unrecognized herb-drug interactions when using combination therapies. Future pharmacokinetic studies are essential to determine whether specific TCM formulations alter the exposure levels of EGFR-TKIs in patients.

### 4.2 Potential mechanisms informed by preclinical evidence

The analysis of syndrome patterns and medication guidelines in this study indicates that for patients with advanced NSCLC undergoing EGFR-TKI therapy, the primary pathomechanisms involve Deficiency of Qi and Yin, Lung-spleen Deficiency. These findings are consistent with the TCM characterization of late-stage lung cancer, which posits that ‘prolonged illness leads to multiple deficiencies, damage to both the Lung and Spleen, and depletion of Qi and Yin (40). Following extended targeted drug therapy aimed at eradicating pathogenic factors, patients often experience a depletion of vital energy and Yin fluids, along with compromised Lung and Spleen functions related to transportation and transformation. This evolution in the disease’s core pathomechanisms underscores the frequent clinical application of tonifying formulas. For addressing the syndrome of concurrent Qi and Yin deficiency, commonly utilized clinical herbs include *Astragali Radix*, *Codonopsis Radix*, *Ophiopogonis Radix*, and *Glehniae Radix*. These herbs are selected for their properties in enhancing Qi, nourishing Yin, consolidating the root, and supporting vital energy, thereby ameliorating the Qi and Yin depletion induced by targeted therapy and restoring the body’s deficiencies.

In addressing secondary pathogenic factors such as Phlegm-Dampness-Stasis, and Blood Stasis that manifest during the progression of the disease, herbs including *Citri Reticulatae Pericarpium*, *Pinelliae Rhizoma*, *Poria*, and *Salvia Chinensis Herba* are employed to fortify the spleen, resolve phlegm, eliminate dampness, and enhance blood circulation (28). This approach targets both symptoms and underlying causes, thereby facilitating the clearance of phlegm-stasis toxins. Herbs commonly used for deficiencies in both the lung and spleen adhere to the ‘strengthen the earth to generate metal’ treatment principle. This involves reinforcing the spleen, tonifying Qi, and consolidating the acquired foundation, which in turn nourishes the lungs through the spleen and stomach. Such interventions improve vital Qi Deficiency and compromised immunity in patients at advanced stages, while also mitigating intolerance to targeted therapies. In cases of heat-toxin obstruction in the lungs, often observed during tumor progression and characterized by intense inflammatory toxins, herbs such as *Hedyotis Diffusae Herba*, *Scutellariae Barbatae Herba*, and *Houttuyniae Herba* are combined to clear heat and detoxify. This combination aids in the removal of stasis toxins from the lungs and provides a supportive role in expelling pathogenic factors and inhibiting tumor growth (41).

In summary, integrating TCM with EGFR-TKI therapy appears to enhance therapeutic efficacy, prolong survival outcomes, and potentially alleviate treatment-related toxicities, thereby improving safety profiles. This combinatorial strategy is largely consistent with the pathological characteristics of the disease and may provide a promising therapeutic paradigm for advanced EGFR-mutant non-small-cell lung cancer. These findings could support future efforts toward standardizing TCM formulations and optimizing relevant treatment regimens.

This study has several limitations that warrant consideration. Firstly, the overall body of evidence is constrained by the inherent limitations of the included trials. These are predominantly small-scale studies exhibiting significant methodological heterogeneity, with certain trials lacking rigor in design and implementation, potentially introducing publication bias. Crucially, while both RCTs and non-randomized studies were included, the findings are primarily driven by RCT evidence. Second, the scope of outcomes assessed was narrow. The analysis focused on short-term efficacy and common adverse reactions, leaving long-term survival benefits unevaluated. Furthermore, substantial clinical heterogeneity exists regarding the application TCM. The included studies lacked standardized guidelines for TCM syndrome differentiation and prescription, preventing meaningful subgroup analyses to determine whether specific herbal formula types (e.g., tonifying vs. detoxifying) yield differential efficacy or detoxifying effects. Therefore, our findings should be interpreted with caution. They underscore the imperative for future high-quality, large-sample, multicenter, double-blind RCTs that adhere to rigorous standards. Such studies should incorporate standardized TCM protocols and include long-term survival and mechanistic biomarkers to provide robust, high-level evidence for integrative TCM therapy.

## 5 Conclusion

In conclusion, the findings of this meta-analysis suggest that the integration of TCM with EGFR-TKIs may offer potential benefits for patients with advanced EGFR-mutant NSCLC, particularly in terms of short-term efficacy and the mitigation of certain adverse events. TCM syndrome differentiation in the included studies primarily identified patterns of Qi and Yin Deficiency, as well as Lung and Spleen Deficiency, with clinical prescriptions aligning with these patterns. It is crucial to emphasize that these results are preliminary and derived from studies with methodological limitations. Therefore, they should be interpreted with caution. The promising potential of this integrated approach warrants confirmation through future, rigorously designed, large-scale, multicenter randomized controlled trials that employ standardized TCM protocols and incorporate mechanistic investigations.

## Data Availability

The original contributions presented in the study are included in the article/**Supplementary Material**. Further inquiries can be directed to the corresponding author.

## References

[1] Thai AA Solomon BJ Sequist LV Gainor JF Heist RS . Lung cancer. Lancet. (2021) 398:535–54. doi: 10.1016/s0140-6736(21)00312-3 34273294

[2] Crinò L Weder W Van Meerbeeck J Felip E . Early stage and locally advanced (non-metastatic) non-small-cell lung cancer: ESMO clinical practice guidelines for diagnosis, treatment and follow-up. Ann Oncol. (2010) 21:v103–15. doi: 10.1093/annonc/mdq207 20555058

[3] Remon J Steuer CE Ramalingam SS Felip E . Osimertinib and other third-generation EGFR TKI in EGFR-mutant NSCLC patients. Ann Oncol. (2018) 29:i20–7. doi: 10.1093/annonc/mdx704 29462255

[4] D'Angelo SP Pietanza MC Johnson ML Riely GJ Miller VA Sima CS . Incidence of EGFR exon 19 deletions and L858R in tumor specimens from men and cigarette smokers with lung adenocarcinomas. J Clin Oncol. (2011) 29:2066–70. doi: 10.1200/JCO.2010.32.6181 PMC3296671 21482987

[5] Midha A Dearden S McCormack R . EGFR mutation incidence in non-small-cell lung cancer of adenocarcinoma histology: a systematic review and global map by ethnicity (mutMapII). Am J Cancer Res. (2015) 5:2892–911. PMC4633915 26609494

[6] Landre T Assié JB Chouahnia K Des Guetz G Auliac JB Chouaïd C . First-line concomitant EGFR-TKI + chemotherapy versus EGFR-TKI alone for advanced EGFR-mutated NSCLC: a meta-analysis of randomized phase III trials. Expert Rev Anticancer Ther. (2024) 24:775–80. doi: 10.1080/14737140.2024.2362889 38813930

[7] Mok TS Wu YL Thongprasert S Yang CH Chu DT Saijo N . Gefitinib or carboplatin-paclitaxel in pulmonary adenocarcinoma. N Engl J Med. (2009) 361:947–57. doi: 10.1056/nejmoa0810699 19692680

[8] Rosell R Carcereny E Gervais R Vergnenegre A Massuti B Felip E . Erlotinib versus standard chemotherapy as first-line treatment for European patients with advanced EGFR mutation-positive non-small-cell lung cancer (EURTAC): a multicentre, open-label, randomised phase 3 trial. Lancet Oncol. (2012) 13:239–46. doi: 10.1016/s1470-2045(11)70393-x 22285168

[9] Lin JJ Cardarella S Lydon CA Dahlberg SE Jackman DM Jänne PA . Five-year survival in EGFR-mutant metastatic lung adenocarcinoma treated with EGFR-TKIs. J Thorac Oncol. (2016) 11:556–65. doi: 10.1016/j.jtho.2015.12.103 26724471 PMC4979601

[10] Wang Z Zhong Z Yang W Li Y Zhang K Gong Y . Traditional Chinese medicines as anticancer agents for non-small cell lung cancer with EGFR mutations: a review. Drug Des Devel Ther. (2025) 19:5169–91. doi: 10.2147/dddt.s522445 40546658 PMC12182731

[11] Feng Y Chen SN Jiang Y Xia Z Chen Y Lin WN . Bufei Huayu decoction combined with gefitinib in the treatment of EGFR mutant advanced non-small cell lung cancer. Chin J Gerontol. (2019) 39:5682–6.

[12] Jiao L Xu J Sun J Chen Z Gong Y Bi L . Chinese herbal medicine combined with EGFR-TKI in EGFR mutation-positive advanced pulmonary adenocarcinoma (CATLA): a multicenter, randomized, double-blind, placebo-controlled trial. Front Pharmacol. (2019) 10:732. doi: 10.3389/fphar.2019.00732 31333456 PMC6614728

[13] Ju LX Han MQ Su JM Zhang Q Tu HB Dong ZY . Clinical study of staged Chinese herbal medicine assisted target therapy in the treatment of non small cell lung cancer with EGFR sensitive mutation. J Shanghai Univ Traditional Chin Med. (2017) 31:18–22. doi: 10.16306/j.1008-861x.2017.03.006

[14] Li CL Hsia TC Yang ST Chao KC Tu CY Chen HJ . Efficacy of prophylactic traditional Chinese medicine on skin toxicity of afatinib in EGFR mutation-positive advanced lung adenocarcinoma: a single-center, prospective, double-blinded, randomized-controlled pilot trial. Integr Cancer Ther. (2022) 21:15347354221086663. doi: 10.1177/15347354221086663 35297709 PMC8943309

[15] Li Y Mou WC Liang P Yu CF . Efficacy and mechanism of modified Wumei pill on diarrhea associated with EGFR-TKI targeted therapy for lung cancer. Clin J Traditional Chin Med. (2022) 34:1122–6. doi: 10.16448/j.cjtcm.2022.0632

[16] Liu CC . Application effect of correcting Qi and eliminating pathogenic factors traditional Chinese medicine combined with targeted therapy in patients with epidermal growth factor receptor mutant non-small cell lung cancer. J Chin Foreign Med Pharm Res. (2026) 5:85–7.

[17] Liu MY . Clinical observation of Bufei Huayu decoction combined with gefitinib in the treatment of EGFR-mutant advanced non-small-cell lung cancer. Contemp Med. (2021) 27:53–5.

[18] Meng JQ Jiao LJ Yao JL Li JQ Gong YB Xu L . Clinical study on Yiqi Yangyin Jiedu decoction combined with icotinib in the treatment of elderly patients with advanced EGFR-mutant lung adenocarcinoma. Modern J Integrated Traditional Chin Western Med. (2022) 31:877–881+1004.

[19] Shen LP Liu LS Jiang Y Gu AQ Wu QQ Zhang Y . Clinical study of Fuzheng Jiedu combined with ectinib hydrochloride in the treatment of advanced lung adenocarcinoma in the elderly. J Tianjin Univ Traditional Chin Med. (2019) 38:445–51.

[20] Tang M Wang S Zhao B Wang W Zhu Y Hu L . Traditional Chinese medicine prolongs progression-free survival and enhances therapeutic effects in epidermal growth factor receptor tyrosine kinase inhibitor (EGFR-TKI) treated non-small-cell lung cancer (NSCLC) patients harboring EGFR mutations. Med Sci Monit. (2019) 25:8430–7. doi: 10.12659/msm.917251 31704907 PMC6865232

[21] Wang Y Wu G Li R Luo Y Huang X He L . Chinese medicine combined with EGFR-TKIs prolongs progression-free survival and overall survival of non-small cell lung cancer (NSCLC) patients harboring EGFR mutations, compared with the use of TKIs alone. Front Public Health. (2021) 9:677862. doi: 10.3389/fpubh.2021.677862 34222178 PMC8249768

[22] Xiong SQ Li YL Wang SM Jin S Luo QY Ding Q . Cohort study of EGFR-TKIs combined with traditional Chinese medicine and single EGFR-TKIs for advanced NSCLC (non-small cell lung cancer. Sichuan Da Xue Xue Bao Yi Xue Ban. (2018) 49:566–569+586. doi: 10.13464/j.scuxbyxb.2018.04.012 30378311

[23] Xu J Zhu YK Guo P Lu JJ Teng WJ Li J . Effect of Fuzheng Quxie recipe combined with targeted therapy on efficacy and immune function in advanced non-small cell lung cancer. Modern J Integrated Traditional Chin Western Med. (2022) 31:1538–41.

[24] Xu RZ Li Y Zhang L Fang ZH Zhao FC Lv X . Clinical and mechanism study of Fuzheng Quxie formula combined with EGFR-TKI in the treatment of lung adenocarcinoma. Anti-tumor Pharm. (2024) 14:723–30.

[25] Yang XB Chai XS Wu WY Long SQ Deng H Pan ZQ . Gefitinib plus Fuzheng Kang'ai formula () in patients with advanced non-small cell lung cancer with epidermal growth factor receptor mutation: a randomized controlled trial. Chin J Integr Med. (2018) 24:734–40. doi: 10.1007/s11655-017-2819-8 28795387

[26] Zhao LC Li LZ Yuan JH Zhuang JN Chen T Fang CT . Clinical study on treatment of advanced non-small cell lung cancer with spleen deficiency and phlegm-damp syndrome by modified Liujunzi decoction combined with gefitinib tablets. J Guangzhou Univ Traditional Chin Med. (2021) 38:250–6. doi: 10.13359/j.cnki.gzxbtcm.2021.02.006

[27] Zhao YY Long SQ Zhou YS Yang XB Liu LR Li QP . Effect of Fuzheng Kang'ai granule and gefitinib on non - small cell lung cancer: a randomized double - blinded controlled trial. Chin J Integrated Traditional West Med. (2020) 40:1310–4.

[28] Li JS Feng ZZ Liu WR . Diagnostic criteria for traditional Chinese medicine syndrome of non-small cell lung cancer(2025). J Traditional Chin Med. (2025) 66:1513–6. doi: 10.13288/j.11-2166/r.2025.14.018

[29] Le X Nilsson M Goldman J Reck M Nakagawa K Kato T . Dual EGFR-VEGF pathway inhibition: a promising strategy for patients with EGFR-mutant NSCLC. J Thorac Oncol. (2021) 16:205–15. doi: 10.1016/j.jtho.2020.10.006 33096270

[30] Yu HA Arcila ME Rekhtman N Sima CS Zakowski MF Pao W . Analysis of tumor specimens at the time of acquired resistance to EGFR-TKI therapy in 155 patients with EGFR-mutant lung cancers. Clin Cancer Res. (2013) 19:2240–7. doi: 10.1158/1078-0432.ccr-12-2246 23470965 PMC3630270

[31] Lu Y Sun C Jiao L Liu Y Gong Y Xu L . Chinese herbal medicine combined with first-generation EGFR-TKIs in treatment of advanced non-small cell lung cancer with EGFR sensitizing mutation: a systematic review and meta-analysis. Front Pharmacol. (2021) 12:698371. doi: 10.3389/fphar.2021.698371 34512332 PMC8429791

[32] Shi Q Jiang Z Yang J Cheng Y Pang Y Zheng N . A flavonoid glycoside compound from Murraya paniculata (L.) interrupts metastatic characteristics of A549 cells by regulating STAT3/NF-κB/COX-2 and EGFR signaling pathways. AAPS J. (2017) 19:1779–90. doi: 10.1208/s12248-017-0134-0 28842850

[33] Yan W Ma X Zhao X Zhang S . Baicalein induces apoptosis and autophagy of breast cancer cells via inhibiting PI3K/AKT pathway *in vivo* and vitro. Drug Des Devel Ther. (2018) 12:3961–72. doi: 10.2147/dddt.s181939 30510404 PMC6248272

[34] Deng M Liu B Song H Yu R Zou D Chen Y . β-Elemene inhibits the metastasis of multidrug-resistant gastric cancer cells through miR-1323/Cbl-b/EGFR pathway. Phytomedicine. (2020) 69:153184. doi: 10.1016/j.phymed.2020.153184 32199253

[35] Li Y Zhou X Niu M Zhang M Gu Q Chen W . Effect of single-dose and short-term administration of Si Jun Zi Tang on the pharmacokinetics of gefitinib in rats. Evid Based Complement Alternat Med. (2021) 2021:6655449. doi: 10.1155/2021/6655449 34354759 PMC8331296

[36] Wu ZH Li MJ Hong YL Chen LX Xu CQ Zhong QW . YiQiChuTan formula (YQCTF) inhibit the progression of non-small cell lung cancer via down-regulating EGFR/ITGB2 signaling: triangulated evidence from network pharmacology, proteomic profiling, and experimental validation. Phytomedicine. (2025) 144:156950. doi: 10.1016/j.phymed.2025.156950 40505482

[37] Xu ZY Li JL . Comparative review of drug-drug interactions with epidermal growth factor receptor tyrosine kinase inhibitors for the treatment of non-small-cell lung cancer. Onco Targets Ther. (2019) 12:5467–84. doi: 10.2147/OTT.S194870 PMC6636179 31371986

[38] Peters S Zimmermann S Adjei AA . Oral epidermal growth factor receptor tyrosine kinase inhibitors for the treatment of non-small cell lung cancer: comparative pharmacokinetics and drug-drug interactions. Cancer Treat Rev. (2014) 40:917–26. doi: 10.1016/j.ctrv.2014.06.010 25027951

[39] Ying Z Wei J Liu R Zhao F Yu Y Tian X . An UPLC-MS/MS method for determination of osimertinib in rat plasma: application to investigating the effect of ginsenoside Rg3 on the pharmacokinetics of osimertinib. Int J Anal Chem. (2020) 2020:8814214. doi: 10.1155/2020/8814214 33456471 PMC7785372

[40] Lee KJ Kwon JH Kim DH Park MS Jin YJ Kim JH . Efficacy of herbal medicine therapy combined with palliative chemotherapy in unresectable advanced NSCLC: a systematic review and meta-analysis. Integr Cancer Ther. (2025) 24:15347354251373045. doi: 10.1177/15347354251373045 41103125 PMC12541183

[41] Xie YG Shen KP Lu YL . Progress in TCM treatment of non-small cell lung cancer. China J Traditional Chin Med Pharm. (2021) 36:2846–51.

