# Supplementary material for: Chinese medicine combined with EGFR-TKIs in EGFR-mutated advanced NSCLC: a meta-analysis of efficacy and safety
Source: Front Oncol. 2026 Oct 12;16:1942222. doi: 10.3389/fonc.2026.1942222 (PMC13696201; doi:10.3389/fonc.2026.1942222)
Supplement: Supplementary file 1 [file Supplementaryfile1.doc]

***Supplementary Materials***

**Supplementary Table 1.The detailed search strategy.**

**Coverage:from the inception to May 18,2026**

| **Database** | **Result** | **Search strings** |
| --- | --- | --- |
| Pubmed | 117 | ((((((((("Medicine,Chinese Traditional"[Mesh])OR(Zhong Yi Xue[Title/Abstract]))OR(Chung I Hsueh[Title/Abstract]))OR(Hsueh,Chung I[Title/Abstract]))OR(Traditional Medicine,Chinese[Title/Abstract]))OR(Chinese Traditional Medicine[Title/Abstract]))OR(Chinese Medicine,Traditional[Title/Abstract]))OR(Chinese Herbal[Title/Abstract]))  AND  (((((((((("Carcinoma,Non-Small-Cell Lung"[Mesh])OR(Carcinomas,Non-Small-Cell Lung[Title/Abstract]))OR(Lung Carcinoma,Non-Small-Cell[Title/Abstract]))OR(Lung Carcinomas,Non-Small-Cell[Title/Abstract]))OR(Non-Small-Cell Lung CarcinomasNon-Small-Cell Lung Carcinomas[Title/Abstract]))OR(Carcinoma,Non-Small Cell Lung[Title/Abstract]))OR(Non-Small Cell Lung Cancer[Title/Abstract]))OR(Non-Small-Cell Lung Carcinoma[Title/Abstract]))OR(Non Small Cell Lung Carcinoma[Title/Abstract]))OR(Nonsmall Cell Lung Cancer[Title/Abstract])))  AND  ((((("Epidermal growth factor receptor"[Mesh])OR(EGFR))OR(EGFR-TKI))OR(exon 19 deletion))OR(exon 21 deletion)) |
| EMBASE | 135 | #1.'Traditional Chinese Medicine'/exp OR'Zhong Yi Xue'/exp OR'Chung I Hsueh'/exp OR'Hsueh,Chung I'/exp OR'Traditional Medicine,Chinese'/exp OR'Chinese Traditional Medicine'/exp OR'Traditional Chinese Medicine'OR'Chinese Medicine,Traditional'OR'Chinese Herbal*'  #2.'Non-small cell lung cancer'/exp OR'Carcinoma,Non Small Cell Lung'/exp OR'Carcinomas,Non-Small-Cell Lung'/exp OR'Lung Carcinoma,Non-Small-Cell':ti,ab OR'Lung Carcinomas,Non-Small-Cell':ti,ab OR'Nonsmall Cell Lung Cancer':ti,ab  #3.'Epidermal growth factor receptor'/exp OR'EGFR'/exp OR'exon 19 deletion'/exp OR'exon 21 deletion'/exp OR'EGFR-TKI*'  #4.#1 AND#2 AND#3 |
| Cochrane Library | 27 | #1 mesh descriptor:[Traditional Chinese Medicine]explode all trees  #2(Zhong Yi Xue or Chung I Hsueh or Hsueh,Chung I or Traditional Medicine or Chinese Traditional Medicine or Chinese Medicine,Traditional or Chinese Herbal):ti,ab,kw(Word variations have been searched)  #3#1 or#2  #4 mesh descriptor:[Non-Small-Cell Lung]explode all trees  #5(Lung Carcinoma,Non-Small-Cell or Lung Carcinomas,Non-Small-Cell or Non-Small-Cell Lung Carcinomas or Carcinoma,Non-Small Cell Lung or Non-Small Cell Lung Cancer or Non-Small-Cell Lung Carcinoma or Non Small Cell Lung Carcinoma or Nonsmall Cell Lung Cancer):ti,ab,kw(Word variations have been searched)  #6#4 or#5  #7 mesh descriptor:[Epidermal growth factor receptor]explode all trees  #8(EGFR or EGFR-TKI or exon 19 deletion or exon 21 deletion):ti,ab,kw(Word variations have been searched)  #9#7 or#8  #10#3 and#6 and#9 |
| Chinese National Knowledge Infrastructure(CNKI) | 355 | (Subject: Traditional Chinese Medicine) OR (Abstract term: Traditional Chinese Medicine + TCM + Chinese herbal medicine + ethnic medicine + Chinese herbal compound formulas + classical TCM prescriptions (exact)) AND (Subject: Non-small cell lung cancer) OR (Abstract term: Non-small cell lung cancer + lung cancer + lung adenocarcinoma + lung squamous cell carcinoma + large cell carcinoma (exact)) AND (Subject: Epidermal growth factor receptor) OR (Abstract term: EGFR + EGFR-TKI inhibitors + exon 19 deletion + exon 21 L858R mutation)) |
| Wanfang Database | 251 | (Topic: (Traditional Chinese Medicine) OR Title or Keywords: (Traditional Chinese Medicine OR Chinese Materia Medica OR Ethnic Medicine OR Chinese Herbal Compound OR Classical Prescriptions of Traditional Chinese Medicine)) AND (Topic: (Non-small Cell Lung Cancer) OR Title or Keywords: (Lung Cancer OR Lung Adenocarcinoma OR Lung Squamous Cell Carcinoma OR Large Cell Carcinoma)) AND (Topic: (Epidermal Growth Factor Receptor) OR Title or Keywords: (EGFR OR EGFR-TKI OR EGFR Tyrosine Kinase Inhibitor OR Exon 19 Deletion OR Exon 21 L858R Mutation)) |
| VIP Database | 278 | Subject = Traditional Chinese Medicine OR Title or Keywords = Traditional Chinese Medicine OR Chinese Materia Medica OR Ethnic Medicine OR Chinese Herbal Compound OR Classical Prescriptions of Traditional Chinese Medicine AND Subject = Non-small Cell Lung Cancer OR Title or Keywords = Lung Cancer OR Lung Adenocarcinoma OR Lung Squamous Cell Carcinoma OR Large Cell Carcinoma AND Subject = Epidermal Growth Factor Receptor OR Title or Keywords = EGFR OR EGFR-TKI OR EGFR Tyrosine Kinase Inhibitor OR Exon 19 Deletion OR Exon 21 L858R Mutation |

**Supplementary Table 2.Characteristics of included studies.**

**Supplementary Table 3.Prescription Information of Included Studies.**

| **Year** | **Author** | **TCM syndrome** | **Herbal Prescription Information** |
| --- | --- | --- | --- |
| 2019 | Tang et al. | Phlegm-dampness-stasis | *Pinelliae Rhizoma*(Banxia)、*Citri Reticulatae Pericarpium*(Chenpi)、*Poria*(Fuling)、*Glycyrrhizae Radix et Rhizoma*(Gancao)、*Pogostemonis Herba*(Guanghuoxiang)、*Armeniacae Semen Amarum*(Kuxingren)、*Talcum*(Huashi)、*Amomi Fructus Rotundus*(Doukou)、*Lophatherum gracile Brongn.*(Danzhuye)、*Coicis Semen*(Yiyiren)、*Magnoliae Officinalis Cortex*(Houpo) |
|  |  | Deficiency for Qi and Yin | *Rhizoma Phragmitis*(Lugen)、*Benincasa hispida*(Dongguazi)、*Persicae Semen*(Taoren)、*Coicis Semen*(Yiyiren)、*Lonicerae Japonicae Flos*(Jinyinhua)、*Houttuyniae Herba*(Yuxingcao)、*Mori Cortex*(Sangbaipi)、*Platycodonis Radix(*Jiegeng) |
|  |  | Heat-toxic obstruction of lung | *Glehniae Radix*(Beishashen)、*Ophiopogonis Radix*(Maidong)、*Schisandrae Chinensis Fructus*(Wuweizi)、*Adenophora stricta*(Shashen)、*Astragali Radix*(Huangqi）、*Anemarrhenae Rhizoma*(Zhimu)、*Ligustri Lucidi Fructus*(Nüzhenzi)、*Paeoniae Radix Alba*(Baishao)、*Atractylodis* *Macrocephalae Rhizoma*(Baizhu)、*Asini Corii Colla*(Ejiao) |
|  |  | Lung-spleen deficiency | *Ginseng Radix et Rhizoma*(Renshen)、*Atractylodis Macrocephalae Rhizoma*(Baizhu)、*Poria*(Fuling)、*Glycyrrhizae Radix et Rhizoma*(Gancao)、*Astragali Radix*(Huangqi）、*Pinelliae Rhizoma*(Banxia)、*Citri Reticulatae Pericarpium*(Chenpi)、*Platycodonis Radix*(Jiegeng)、*Armeniacae Semen Amarum*(Kuxingren) |
| 2021 | Wang et al. | Qi Deficiency | *Astragali Radix*(Huangqi）、*Ginseng Radix et Rhizoma*(Renshen)、*Atractylodis Macrocephalae Rhizoma*(Baizhu) |
|  |  | Yin Deficiency with Lung Dryness | *Astragali Radix*(Huangqi）、*Ginseng Radix et Rhizoma*(Renshen)、*Atractylodis Macrocephalae Rhizoma*(Baizhu)、*Armeniacae Semen Amarum*(Kuxingren)、*Platycodonis Radix*(Jiegeng) |
|  |  | Phlegm-dampness-stasis | *Astragali Radix*(Huangqi）、*Ginseng Radix et Rhizoma*(Renshen)、*Atractylodis Macrocephalae Rhizoma*(Baizhu)、*Pinelliae Rhizoma*(Banxia)、*Citri Reticulatae Pericarpium*(Chenpi) |
|  |  | Phlegm-dampness-stasis | *Astragali Radix*(Huangqi）、*Ginseng Radix et Rhizoma*(Renshen)、*Atractylodis Macrocephalae Rhizoma*(Baizhu)、*Coptidis Rhizoma*(Huanglian) |
|  |  | Yin Deficiency | *Astragali Radix*(Huangqi）、*Ginseng Radix et Rhizoma*(Renshen)、*Atractylodis Macrocephalae Rhizoma*(Baizhu)、*Ophiopogonis Radix*(Maidong)、*Adenophora stricta*(Shashen) |
|  |  | Lung-spleen deficiency | *Astragali Radix*(Huangqi）、*Ginseng Radix et Rhizoma*(Renshen)、*Atractylodis Macrocephalae Rhizoma*(Baizhu)、*Lonicerae Japonicae Flos*(Jinyinhua)、*Forsythiae Fructus*(Lianqiao)、*Phellodendri Chinensis Cortex*(Huangbo) |
| 2022 | Li(1)et al. | Yin Deficiency | *Rehmanniae Radix Cortex*(Dihuang)、*Rehmanniae Radix Cortex*(Dihuang)、*Ophiopogonis Radix*(Maidong)、*Lilium lancifolium*(Baihe)、*Paeoniae Radix Alba*(Baishao)、*Angelicae Sinensis Radix*(Danggui)、*Fritillariae Chrrhosae Bulbus*(Chuanbeimu)、*Glycyrrhizae Radix et Rhizoma*(Gancao)、*Scrophulariae Radix*(Xuanshen)、*Platycodonis Radix*(Jiegeng) |
|  |  | Heat-toxic obstruction of lung | *Pinelliae Rhizoma*(Banxia)、*Bambusae Caulis In Taenias*(Zhuru)、*Aurantii Fructus Immaturus*(Zhuru)、*Citri Reticulatae Pericarpium*(Chenpi)、*Zingiber officinale Roscoe*(Shenjiang)、*Glycyrrhizae Radix et Rhizoma*(Gancao)、*Poria*(Fuling)、*Jujubae Fructus*(Dazao) |
|  |  | Heat-toxic obstruction of lung | *Saposhnikoviae Radix*(Fangfeng)、*Forsythiae Fructus*(Lianqiao)、*Angelicae Dahuricae Radix*(Baizhi)、*Platycodonis Radix*(Jiegeng)、*Scutellariae Radix*(Huangqin)、*Chuanxiong Rhizoma*(Chuanxiong)、*Coptidis Rhizoma*(Huanglian)、*Schizonepetae Herba*(Jingjie)、*Gardeniae Fructus*(Zhizi)、*Aurantii Fructus*(Zhiqiao)、*Menthae Haplocalycis Herba*(Bohe)、*Glycyrrhizae Radix et Rhizoma*(Gancao) |
| 2022 | Xu(1)et al. | Deficiency for Qi and Yin | *Astragali Radix*(Huangqi）、*Atractylodis Macrocephalae Rhizoma*(Baizhu)、*Ophiopogonis Radix*(Maidong)、*Poria*(Fuling)、*Citri Reticulatae Pericarpium*(Chenpi)、*Asparagi Radix*(Tiandong)、*Glehniae Radix*(Beishashen)、*Selaginellaceae*(Shishangbai)、*Salvia Chinensis Herba*(Shijianchuan)、*Hedyotis Diffusae Herba*(Baihuasheshecao)、*Scutellariae Barbatae Herba*(Banzhilian) |
| 2026 | Liu et al. | Lung-spleen deficiency | *Astragali Radix*(Huangqi）、*Codonopsis Radix*(Dangshen)、*Atractylodis Macrocephalae Rhizoma*(Baizhu)、*Poria*(Fuling)、*Ganoderma*(Lingzhi)、*Salviae Miltiorrhizae Radix et Rhizoma*(Danshen)、*Lilium lancifolium*(Baihe)、*Anemarrhenae Rhizoma*(Zhimu)、*Ophiopogonis Radix*(Maidong)、*Aurantii Fructus Immaturus*(Zhuru)、*Citri Reticulatae Pericarpium*(Chenpi) |
| 2022 | Xu(2)et al. | Deficiency for Qi and Yin | *Astragali Radix*(Huangqi）、*Atractylodis Macrocephalae Rhizoma*(Baizhu)、*Poria*(Fuling)、*Glehniae Radix*(Beishashen)、*Asparagi Radix*(Tiandong)、*Ophiopogonis Radix*(Maidong)、*Salvia Chinensis Herba*(Shijianchuan)、*Selaginellaceae*(Shishangbai)、*Hedyotis Diffusae Herba*(Baihuasheshecao)、*Prunellae Spica*(Xiakucao)、*Sargassum*(Haizao)、*Laminariae Thallus*(Kunbu) |
| 2018 | Xiong et al. | Phlegm-dampness-stasis | *Citri Reticulatae Pericarpium*(Chenpi)、*Pogostemonis Herba*(Guanghuoxiang)、*Pinelliae Rhizoma*(Banxia)、*Poria*(Fuling)、*Armeniacae Semen Amarum*(Kuxingren)、*Talcum*(Huashi)、*Tetrapanacis Medulla*(Tongcao)、*Amomi Fructus Rotundus(*Doukou)、*Lophatherum gracile Brongn.*(Danzhuye)、*Magnoliae Officinalis Cortex*(Houpo)、*Coicis Semen*(Yiyiren) |
|  |  | Deficiency for Qi and Yin | *Pseudostellariae Radix*(Taizishen)、*Ophiopogonis Radix*(Maidong)、*Schisandrae Chinensis Fructus*(Wuweizi)、*Adenophora stricta*(Shashen)、*Anemarrhenae Rhizoma*(Zhimu)、*Astragali Radix*(Huangqi）、*Ligustri Lucidi Fructus*(Nüzhenzi)、*Ginseng Radix et Rhizoma*(Renshen)、*Paeoniae Radix Alba*(Baishao)、*Angelicae Sinensis Radix*(Danggui)、*Atractylodis Macrocephalae Rhizoma*(Baizhu)、*Asini Corii Colla*(Ejiao)、*Artemisiae Annuae Herba*(Qinghao)、*Trionycis Carapax*(Biejia)、*Phellodendri Chinensis Cortex*(Huangbo) |
|  |  | Heat-toxic obstruction of lung | *Rhizoma Phragmitis*(Lugen)、*Coicis Semen*(Yiyiren)、*Benincasa hispida*(Dongguazi)、*Persicae Semen*(Taoren)、*Lonicerae Japonicae Flos*(Jinyinhua)、*Houttuyniae Herba*(Yuxingcao)、*Platycodonis Radix*(Jiegeng)、*Mori Cortex*(Sangbaipi) |
|  |  | Lung-spleen deficiency | *Astragali Radix*(Huangqi）、*Ginseng Radix et Rhizoma*(Renshen)、*Atractylodis Macrocephalae Rhizoma*(Baizhu)、*Poria*(Fuling)、*Pinelliae Rhizoma*(Banxia)、*Citri Reticulatae Pericarpium*(Chenpi)、*Armeniacae Semen Amarum*(Kuxingren)、*Platycodonis Radix*(Jiegeng)、*Schisandrae Chinensis Fructus*(Wuweizi)、*Lablab Semen Album*(Baibiandou)、*Polygonati Rhizom*a(Huangjing)、*Coicis Semen*(Yiyiren)、*Amomi Fructus Rotundus*(Doukou) |
| 2020 | Zhao et al. | Deficiency for Qi and Yin | *Pseudostellariae Radix*(Taizishen)、*Atractylodis Macrocephalae Rhizoma(*Baizhu)、*Astragali Radix*(Huangqi）、*Hedyotis Diffusae Herba*(Baihuasheshecao)、*Solanum nigrum L.*(Longkui)、*Salvia Chinensis Herba*(Shijianchuan)、*Pleiones Pseudobulbus*(Shancigu)、*Coicis Semen*(Yiyiren)、*Spergularia rubra*(Bayueli)、*Rubus parvifolius*(Shepaole)、*Curcumae Rhizoma*(Ezhu)、*Glycyrrhizae Radix et Rhizoma*(Gancao) |
| 2018 | Yang(1)et al. | Lung-spleen deficiency | *Glehniae Radix*(Beishashen)、*Atractylodis Macrocephalae Rhizoma*(Baizhu)、*Astragali Radix*(Huangqi）、*Hedyotis Diffusae Herba*(Baihuasheshecao)、*Solanum nigrum L.*(Longkui)、*Dendrobii Caulis*(Shihu)、*Rehmanniae Radix Cortex*(Dihuang)、*Coicis Semen*(Yiyiren)、*Schisandrae Chinensis Fructus*(Wuweizi)、*Rubi Fructus*(Fupenzi)、*Curcumae Longae Rhizoma*(Jianghuang)、*Glycyrrhizae Radix et Rhizoma*(Gancao) |
| 2021 | Liu et al. | Qi Deficiency | *Codonopsis Radix*(Dangshen)、*Rehmanniae Radix Cortex*(Dihuang)、*Hedyotis Diffusae Herba*(Baihuasheshecao)、*Paeoniae Radix Rubra*(Chishao)、*Chuanxiong Rhizoma*(Chuanxiong)、*Asteris Radix et Rhizoma*(Ziwan)、*Salviae Miltiorrhizae Radix et Rhizoma*(Danshen)、*Armeniacae Semen Amarum*(Kuxingren)、*Sparganii Rhizoma*(Sanleng)、*Peucedani Radix*(Qianhu)、*Curcumae Rhizoma*(Ezhu)、*Scutellariae Barbatae Herba*(Banzhilian)、*Trionycis Carapax*(Biejia)、*Glycyrrhizae Radix et Rhizoma*(Gancao) |
| 2019 | Shen et al. | Yin Deficiency | *Adenophora stricta*(Shashen)、*Glehniae Radix*(Beishashen)、*Ophiopogonis Radix*(Maidong)、*Asparagi Radix*(Tiandong)、*Lilium lancifolium*(Baihe)、*Prunellae Spica*(Xiakucao)、*Ostreae Concha*(Muli)、*Selaginellaceae*(Shishangbai)、*Salvia Chinensis Herba*(Shijianchuan)、*Hedyotis Diffusae Herba*(Baihuasheshecao) |
|  |  | Qi Deficiency | *Astragali Radix*(Huangqi）、*Codonopsis Radix*(Dangshen)、*Atractylodis Macrocephalae Rhizoma*(Baizhu)、*Poria*(Fuling)、*Epimedii Folium*(Yinyanghuo)、*Prunellae Spica*(Xiakucao)、*Ostreae Concha*(Muli)、*Selaginellaceae*(Shishangbai)、*Salvia Chinensis Herba*(Shijianchuan)、*Hedyotis Diffusae Herba*(Baihuasheshecao) |
|  |  | Deficiency for Qi and Yin | *Astragali Radix*(Huangqi）、*Atractylodis Macrocephalae Rhizoma*(Baizhu)、*Glehniae Radix*(Beishashen)、*Asparagi Radix*(Tiandong)、*Ligustri Lucidi Fructus*(Nüzhenzi)、*Lycii Fructus*(Gouqizi)、*Prunellae Spica*(Xiakucao)、*Ostreae Concha*(Muli)、*Selaginellaceae*(Shishangbai)、*Salvia Chinensis Herba*(Shijianchuan)、*Hedyotis Diffusae Herba*(Baihuasheshecao) |
| 2019 | Feng et al. | Qi Deficiency and Blood Stasis | *Codonopsis Radix*(Dangshen)、*Astragali Radix*(Huangqi）、*Rehmanniae Radix Cortex*(Dihuang)、*Asteris Radix et Rhizoma*(Ziwan)、*Chuanxiong Rhizoma*(Chuanxiong)、*Paeoniae Radix Rubra*(Chishao)、*Salviae Miltiorrhizae Radix et Rhizoma*(Danshen)、*Peucedani Radix*(Qianhu)、*Armeniacae Semen Amarum*(Kuxingren)、*Curcumae Rhizoma*(Ezhu)、*Sparganii Rhizoma*(Sanleng)、*Scutellariae Barbatae Herba*(Banzhilian)、*Hedyotis Diffusae Herba*(Baihuasheshecao)、*Trionycis Carapax*(Biejia)、*Glycyrrhizae Radix et Rhizoma*(Gancao) |
| 2021 | Zhao et al. | Lung-spleen deficiency | *Codonopsis Radix*(Dangshen)、*Atractylodis Macrocephalae Rhizoma*(Baizhu)、*Poria*(Fuling)、*Glycyrrhizae Radix et Rhizoma*(Gancao)、*Citri Reticulatae Pericarpium*(Chenpi)、*Pinelliae Rhizoma*(Banxia) |
| 2017 | Ju et al. | Deficiency for Qi and Yin | *Astragali Radix*(Huangqi）、*Atractylodis Macrocephalae Rhizoma*(Baizhu)、*Poria*(Fuling)、*Angelicae Sinensis Radix*(Danggui)、*Agrimoniae Herba*(Xianhecao)、*Houttuyniae Herba*(Yuxingcao)、*Benincasa hispida*(Dongguazi)、*Coicis Semen*(Yiyiren)、*Citri Reticulatae Pericarpium*(Chenpi)、*Chuanxiong Rhizoma*(Chuanxiong)、*Salvia Chinensis Herba*(Shijianchuan)、*Hedyotis Diffusae Herba*(Baihuasheshecao)、*Glycyrrhizae Radix et Rhizoma*(Gancao) |
|  |  | Deficiency for Qi and Yin | *Astragali Radix*(Huangqi）、*Ophiopogonis Radix*(Maidong)、*Asparagi Radix*(Tiandong)、*Glehniae Radix*(Beishashen)、*Dioscoreae Rhizoma*(Shanyao)、*Ligustri Lucidi Fructus*(Nüzhenzi)、*Ecliptae Herba*(Mohanlian)、*Salvia Chinensis Herba*(Shijianchuan)、*Hedyotis Diffusae Herba*(Baihuasheshecao)、*Setariae Germinatus Fructus*(Guya)、*Fructus Hordei Germinatus*(Maiya)、*Crataegi Fructus*(Shanzha) |
| 2022 | Meng et al. | Qi Deficiency | *Astragali Radix*(Huangqi）、*Codonopsis Radix*(Dangshen)、*Atractylodis Macrocephalae Rhizoma*(Baizhu)、*Poria*(Fuling)、*Prunellae Spica*(Xiakucao)、*Arisaematis Rhizoma*(Tiannanxing)、*Selaginellaceae*(Shishangbai)、*Salvia Chinensis Herba*(Shijianchuan) |
|  |  | Yin Deficiency | *Adenophora stricta*(Shashen)、*Glehniae Radix*(Beishashen)、*Asparagi Radix*(Tiandong)、*Ophiopogonis Radix*(Maidong)、*Prunellae Spica(*Xiakucao)、*Arisaematis Rhizoma*(Tiannanxing)、*Selaginellaceae*(Shishangbai)、*Salvia Chinensis Herba*(Shijianchuan) |
|  |  | Deficiency for Qi and Yin | *Adenophora stricta*(Shashen)、*Glehniae Radix*(Beishashen)、*Asparagi Radix*(Tiandong)、*Ophiopogonis Radix*(Maidong)、*Prunellae Spica*(Xiakucao)、*Arisaematis Rhizoma*(Tiannanxing)、*Selaginellaceae*(Shishangbai)、*Salvia Chinensis Herba*(Shijianchuan) |

**Supplementary Table 4. Risk of bias of all included cohort studies using the Newcastle-Ottawa Scale.**

| **Author** | **Year** | **Selection** | | | | **Comparability** | **Outcome** | | | **Total score** | **Risk level^#^** |
| --- | --- | --- | --- | --- | --- | --- | --- | --- | --- | --- | --- |
|  |  | **Representativenes s of the Exposed Cohort** | **Selection of the Non-Exposed Cohort** | **Ascertainment of Exposure** | **Demonstration That Outcome of Interest Was Not Present at Start of Study** |  | **Assessment of Outcome** | **Was Follow-Up Long Enough for Outcomes to Occur** | **Adequacy of Follow Up of Cohorts** |  |  |
| Tang et al. | 2019 | 1 | 1 | 1 | 0 | 0 | 1 | 0 | 1 | 6 | moderate |
| Wang et al. | 2021 | 1 | 0 | 1 | 1 | 1 | 0 | 1 | 1 | 6 | moderate |
| Xu(1)et al. | 2022 | 1 | 1 | 0 | 0 | 1 | 0 | 1 | 1 | 5 | moderate |
| Xiong et al. | 2018 | 1 | 0 | 0 | 0 | 1 | 1 | 1 | 1 | 5 | moderate |

^#^Low(total score≥7),moderate(total score 5-6),and high(total score≤4)risk of bias.

**Supplementary Table 5. GRADE evidence‑certainty assessment for critical and important outcomes of TCM combined with EGFR‑TKI vs. EGFR‑TKI alone in EGFR‑mutant non‑small‑cell lung cancer**

| **Certainty assessment** | | | | | | | **№ of patients** | | **Effect** | | **Certainty** | | **Importance** |
| --- | --- | --- | --- | --- | --- | --- | --- | --- | --- | --- | --- | --- | --- |
| **№ of studies** | **Study design** | **Risk of bias** | **Inconsistency** | **Indirectness** | **Imprecision** | **Other considerations** | **Intervention** | **Control** | **Relative (95% CI)** | **Absolute (95% CI)** |  |  |  |
| **mPFS** | | | | | | | | | | | | | |
| 11 | randomised trials | serious^a^ | not serious | not serious | not serious | none | -/0 | -/0 | **HR 0.70** (0.65 to 0.76) | **1 fewer per 1,000** (from 1 fewer to 1 fewer) | ⨁⨁⨁◯ Moderate^a^ | | CRITICAL |
| **EGFR - Exon 19 deletion** | | | | | | | | | | | | | |
| 4 | randomised trials | serious^a^ | not serious | not serious | not serious | none | -/0 | -/0 | **HR 0.64** (0.51 to 0.79) | **1 fewer per 1,000** (from 1 fewer to 1 fewer) | ⨁⨁⨁◯ Moderate^a^ | | CRITICAL |
| **EGFR - Exon 21 L858R mutation** | | | | | | | | | | | | | |
| 5 | randomised trials | serious^a^ | serious^b^ | not serious | not serious | none | -/0 | -/0 | **HR 0.60** (0.46 to 0.78) | **1 fewer per 1,000** (from 1 fewer to 0 fewer) | ⨁⨁◯◯ Low^a,b^ | | CRITICAL |
| **DCR** | | | | | | | | | | | | | |
| 8 | randomised trials | serious^a^ | serious^c^ | not serious | serious^d^ | none | 481/533 (90.2%) | 362/466 (77.7%) | **RR 1.19** (1.01 to 1.40) | **148 more per 1,000** (from 8 more to 311 more) | ⨁◯◯◯ Very low^a,c,d^ | | CRITICAL |
| **Rashes** | | | | | | | | | | | | | |
| 15 | randomised trials | serious^a^ | serious^c^ | not serious | not serious | none | 345/1022 (33.8%) | 442/918 (48.1%) | **RR 0.75** (0.61 to 0.92) | **120 fewer per 1,000** (from 188 fewer to 39 fewer) | ⨁⨁◯◯ Low^a,c^ | IMPORTANT | |
| **Diarrhea** | | | | | | | | | | | | | |
| 12 | randomised trials | serious^a^ | serious^b^ | not serious | not serious | none | 216/744 (29.0%) | 241/641 (37.6%) | **RR 0.66** (0.51 to 0.86) | **128 fewer per 1,000** (from 184 fewer to 53 fewer) | ⨁⨁◯◯ Low^a,b^ | IMPORTANT | |
| **ALT/AST increasing** | | | | | | | | | | | | | |
| 7 | randomised trials | serious^a^ | not serious | not serious | not serious | none | 36/392 (9.2%) | 46/310 (14.8%) | **RR 0.61** (0.41 to 0.91) | **58 fewer per 1,000** (from 88 fewer to 13 fewer) | ⨁⨁⨁◯ Moderate^a^ | IMPORTANT | |
| **Dental ulcer** | | | | | | | | | | | | | |
| 5 | randomised trials | serious^a^ | not serious | not serious | serious^d^ | none | 15/257 (5.8%) | 25/175 (14.3%) | **RR 0.42** (0.23 to 0.80) | **83 fewer per 1,000** (from 110 fewer to 29 fewer) | ⨁⨁◯◯ Low^a,d^ | IMPORTANT | |

**CI:** confidence interval; **HR:** hazard ratio; **RR:** risk ratio

#### Explanations

a. Most of the included RCTs did not report allocation concealment and blinding implementation processes

b. There is moderate statistical heterogeneity; the point‑estimate effects of individual studies show certain fluctuations, and the confidence intervals of some studies cross the line of no effect.

c. There is substantial heterogeneity; the point‑estimate effects vary widely across studies, the confidence intervals of some studies cross the line of no effect, and there is marked clinical and statistical heterogeneity.

d. Pooled effect 95% CI 1.01‑1.40, the lower limit of CI is very close to the line of no effect (RR=1); the overall effect P=0.04, at the borderline statistical significance level, with a wide confidence interval and imprecise effect estimate.

**Supplementary Figure 1.** Forest plot comparing TCM+EGFR-TKI and EGFR-TKI treatments for DCR in patients with EGFR mutation-positive advanced NSCLC, excluding two studies.

**Supplementary Figure 2. Four properties,five flavors,and meridian tropism of the included Chinese medicinals**

|  |  |  |
| --- | --- | --- |

**Supplementary Figure 3. Bias analysis.**

**Supplementary Figure 4. Sensitivity analysis.**

Note.PFS comparison between TCM combined with EGFR-TKI and EGFR-TKI alone.

**Supplementary Figure 5. Sensitivity analysis.**

Note.PFS comparison between TCM combined with EGFR-TKI and EGFR-TKI alone.The results shown are the culling of studies from Liu *et al.*

**Supplementary Figure 6. Publication bias.**

|  |  |
| --- | --- |

Note.The publication bias of studies on PFS comparison between TCM combined with EGFR-TKI and EGFR-TKI alone.
